# A Treatise on Fever

**Published:** 1861-03

**Authors:** 


A Treatise on Fever; its Cause, Phenomena, and Treatment. With an Appendix containing views on some Female Diseases, some Diseases of Children, &c. By Rezin Thompson, M. D., Nashville, Tenn. 1860.

This is a title to a small octavo volume of 440 pages. It is published in fair style by the author; but the style in which it is written, and its pretensions are certainly unique, and amenable to severe criticism. We hardly think the importance of the work, however, sufficient to justify the expenditure of so much time and space as would be necessary for a serious criticism. The chief substance; the special pith of the book, may be stated in a few words.

The author adopts the well known neurological pathology of Fevers, referring the first link in the chain of morbid action to a morbid impression on the nervous system. He intimates a belief that this morbid impression, is produced by poisonous animalcula. As an antidote or a specific remedy for these animalcula, or whatever other poison may cause fever, he recommends the *oil of Sassafras*, with as much confidence as the noted Samuel Thompson ever did his Lobelia and Steam. On this point we quote his language as follows:

"Having come to the conclusion that the cause of all idiopathic fevers is a something possessing the property of a narcotic poison, I believed that the oil of sassafras would destroy or counteract, or neutralize it. I had tried it for these purposes a thousand times, successfully, in other analogous cases. For example: I had tried it repeatedly for destroying insect life, and found it immediately fatal to every kind; had also tested its powers upon the infusoria in impure water, and saw it destroy them instantly. I therefore felt assured that if the cause was really the presence of minute animalcules disturbing the nervous centres, this would be conveyed by the circulation in sufficient strength to destroy them at once I also knew that it would as certainly destroy or neutralize any poison which they might have infused into the system, for I had tested its power fully in destroying the poison of insects and reptiles, such as mosquitoes, fleas, spiders, bees, wasps, etc.; and, on one occasion, had an opportunity of testing its powers over the venom of the snake known as the copper-head, and found it to succeed promptly. I also knew, from repeated experiments, that it would destroy or prevent the effects of vegetable narcotics, such as tobacco, henbane, etc.; I therefore judged that, should I be mistaken in the cause of fevers being animalcules, it must of course, then, be a poisonous gas; and, believing, from observing its effects, that it must be of the narcotic kind, I had good reason to infer that the oil of sassafras would succeed, should this be the case. I therefore expected it would meet the first indication, and was not disappointed. I have seen its exhibition followed, again and again, with as sudden a disappearance of the febrile movement as I ever saw follow the opening of an abscess, when the fever originated from that cause."

His favorite mode of prescribing the oil, is in the following combination, which he calls "Fever Syrup:"

| | | |
|---|---|---|
| ℞ | Syrup of Rheubarb, | ℥jv. |
| | Tinct. Valerian, | ℥j. |
| | Oil of Sassafras, | 20 gtts. |
| | Piperin, | 10 grs. |
| | Supr. Carbonate of Soda, | 20 grs. |

Mix. Dose, a table-spoonful every two or three hours.

---

We have received, through the Book Store of W. B. Keen & Co.; Lake Street, Chicago; a copy of the Transactions of the American Medical Association for 1860; Lyons on Fevers; and Dalton's Human Phisiology; which we shall endeavor to notice more fully in our next issue.

